# The role of Rad51 in safeguarding mitochondrial activity during the meiotic cell cycle in mammalian oocytes

**DOI:** 10.1038/srep34110

**Published:** 2016-09-28

**Authors:** Kyeoung-Hwa Kim, Ji-Hoon Park, Eun-Young Kim, Jung-Jae Ko, Kyung-Soon Park, Kyung-Ah Lee

**Affiliations:** 1 Institute of Reproductive Medicine, Department of Biomedical Science, College of Life Science, CHA University, Pangyo-Ro 335, Bundang-gu, Seongnam-si, Gyeonggi-do, 463-400, Korea

## Abstract

Rad51 is a conserved eukaryotic protein that mediates the homologous recombination repair of DNA double-strand breaks that occur during mitosis and meiosis. In addition, Rad51 promotes mitochondrial DNA synthesis when replication stress is increased. Rad51 also regulates cell cycle progression by preserving the G2/M transition in embryonic stem cells. In this study, we report a novel function of Rad51 in regulating mitochondrial activity during *in vitro* maturation of mouse oocytes. Suppression of Rad51 by injection of *Rad51* dsRNA into germinal vesicle-stage oocytes resulted in arrest of meiosis in metaphase I. *Rad51*-depleted oocytes showed chromosome misalignment and failures in spindle aggregation, affecting the completion of cytokinesis. We found that *Rad51* depletion was accompanied by decreased ATP production and mitochondrial membrane potential and increased DNA degradation. We further demonstrated that the mitochondrial defect activated autophagy in *Rad51*-depleted oocytes. Taken together, we concluded that Rad51 functions to safeguard mitochondrial integrity during the meiotic maturation of oocytes.

Homologous recombination (HR) is the most prominent repair pathway for DNA double-strand breaks (DSBs). The central steps of HR involve the processing of DSB ends, a homology search and strand invasion. Efficient repair of DNA DSBs by HR depends on the formation of a Rad51 recombinase filament of broken single-stranded DNA (ssDNA) that facilitates strand invasion between the ssDNA and homologous double-strand DNA (dsDNA)^1^,^2^. If DNA DSBs are not properly repaired, they induce chromosome remodeling, cell cycle arrest or apoptosis. Because Rad51 performs an important function in HR, it protects genome integrity and enables cells to resist apoptosis triggered by DNA damage^3^,^4^,^5^. Additionally, Rad51 facilitates replication fork progression by preventing replication fork collapse, thereby ensuring cell proliferation in vertebrates^6^,^7^. In mouse embryonic stem cells, Rad51 regulates cell cycle progression by preserving the G2/M transition^8^.

Oocytes display a unique cell cycle progression during growth and meiotic maturation. In mammalian oocytes, meiosis generates haploid gametes after one round of chromosomal replication and two successive divisions, meiosis I and meiosis II. Oocytes have two points of arrest in the meiotic cell cycle, during the diplotene stage of prophase I and during metaphase II (MII), which both occur prior to fertilization with sperm. Several reports have shown that HR and Rad51 play important roles in ensuring genome stability in oocytes by repairing DNA damage that is either chemically induced or age related^3^,^9^,^10^,^11^,^12^,^13^. Rad51 expression shows an age-dependent decrease in mouse oocytes and is closely linked to increased aneuploidy rates^11^,^12^. Microinjection of Rad51 into oocytes of AKR/J mice also suppresses apoptosis and reduces the extent of DNA DSBs^13^.

Oocyte meiotic maturation involves cytoplasm maturation as well as nuclear maturation. Nuclear maturation includes chromosome segregation, and cytoplasm maturation is associated with the dynamics of various cytoplasmic components, including membranous organelles. *In vitro* maturation analysis revealed that DNA damage has differential effects on oocyte maturation depending on the degree of the damage^14^,^15^,^16^,^17^. Upon slight DNA DSBs, oocytes from fully grown mice can undergo meiosis resumption as indicated by germinal vesicle breakdown (GVBD)^17^. However, oocytes with severe DNA DSBs do not extrude the first polar body (PB) and cannot reach the MII stage during *in vitro* maturation^17^. A negative correlation between degree of DSBs and embryo developmental capacity has also been reported^18^. Another study focused on the role of Rad51 in the meiotic maturation of undamaged oocytes^19^. In a mouse model carrying a hypomorphic allele of Rad51, which overcomes the early embryonic lethality of the Rad51 null allele, oocytes displayed precocious separation of sister chromatids, aneuploidy and broken chromosomes at MII, possibly due to defects in sister chromatid cohesion at MII^19^. However, the molecular mechanisms underlying Rad51 activity during the meiotic maturation of oocytes remain to be elucidated.

In this study, we report for the first time that Rad51 regulates mitochondrial activity during *in vitro* maturation of mouse oocytes. Oocytes went into MI arrest following the suppression of Rad51 during *in vitro* oocyte maturation. We found that *Rad51* depletion led to reductions in ATP production, mitochondrial potential, and mitochondrial quantity in MI-stage oocytes, supporting a novel role of Rad51 in the maintenance of mitochondrial dynamics and activity during the meiotic cell cycle in oocytes.

## Results

### Silencing of Rad51 in GV oocytes resulted in MI arrest

**Figure 1:**
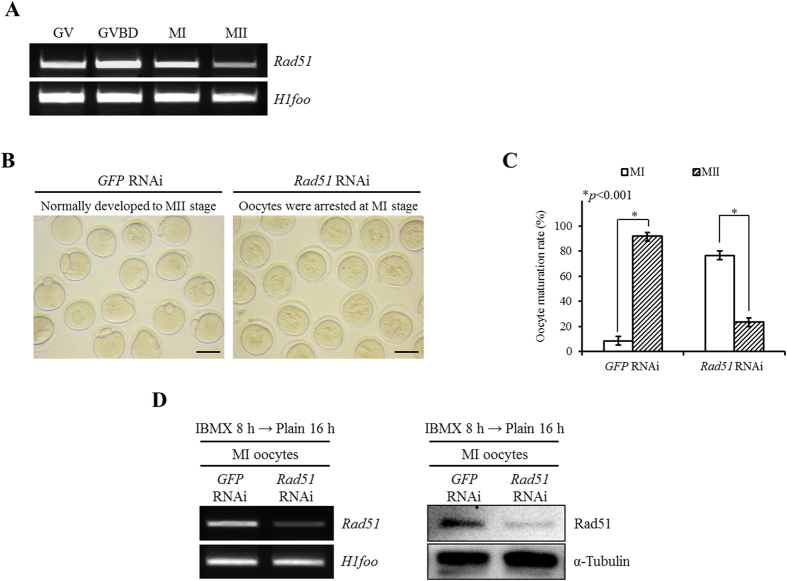
Microinjection of *Rad51* dsRNA into the cytoplasms of GV oocytes resulted in cell arrest at the MI stage. (**A**) Typical pattern of *Rad51* mRNA expression during oocyte maturation. For the PCR reaction, a single oocyte-equivalent mRNA was used as a template for the amplification of each gene. GV-, GVBD-, MI- and MII-stage oocytes were harvested after *in vitro* culture for 0, 2, 8 and 16 hours, respectively. *H1foo* was used as an internal control. (**B**) Micrographs of oocytes treated with *GFP* RNAi as a control or with *Rad51* RNAi. The scale bars indicate 100 μm. *GFP* RNAi, *GFP* dsRNA-injected MII oocyte; *Rad51* RNAi, *Rad51* dsRNA-injected MI oocyte. (**C**) *In vitro* maturation rates of mouse oocytes after microinjection with *Rad51* or *GFP* dsRNA into GV-stage oocytes. The asterisks represent statistical significance at *p* < 0.001. (**D**) *Rad51* RNAi treatment resulted in specific suppression of *Rad51* mRNA (left panel) and protein (right panel) expression. Protein lysate from 150 MI oocytes was loaded into each lane. *H1foo* and α-Tubulin were used as internal controls for mRNA and protein expression, respectively. IBMX 8 h → Plain 16 h, *Rad51*-injected oocytes cultured in IBMX supplement for 8 hours followed by 16 hours of culture in plain M16 medium.

We first examined the expression level of *Rad51* during oocyte maturation. As shown in Fig. 1A, *Rad51* transcripts were expressed throughout meiosis. Specifically, the expression of *Rad51* mRNA was maintained from the GV to the MI stage and reduced at the MII stage (Fig. 1A). In the control group, the *GFP* dsRNA-injected oocytes completed the meiosis process and developed normally to the MII stage with emission of first PBs (91.49%; Fig. 1B,C, Table 1, Video S1). In contrast, based on morphology, 76.48% of the *Rad51*-silenced oocytes were arrested at the MI stage, and 23.52% of the oocytes showed the first PB extrusion (Fig. 1B,C, Table 1, Video S2). We confirmed that there were reductions in Rad51 mRNA and protein levels in the MI-arrested oocytes after treatment with *Rad51* RNAi (Fig. 1D).

### Silencing of Rad51 resulted in increased DNA damage during meiosis

**Figure 2:**
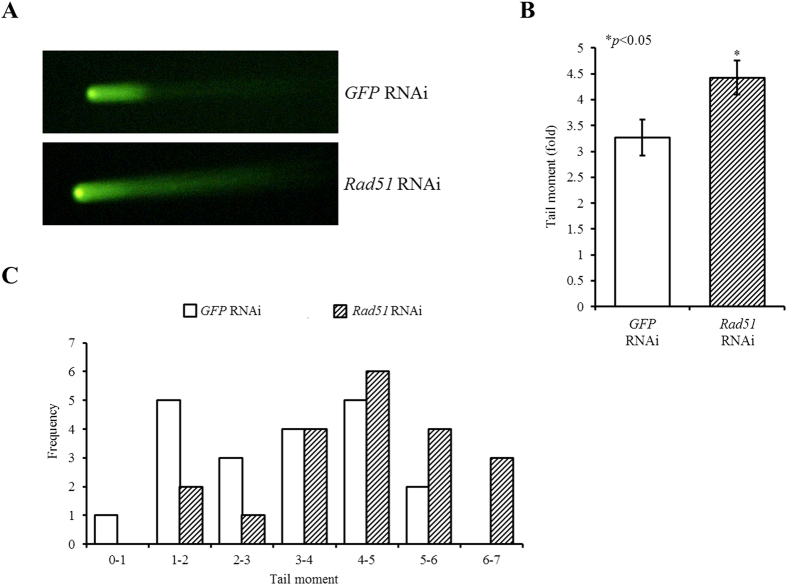
Inhibition of *Rad51* expression induced DNA damage in oocytes. (**A**) *Rad51*-silenced MI oocytes were assessed for DNA damage using a comet assay. *GFP* dsRNA-injected MI oocytes exhibited slight DNA damage, whereas *Rad51*-silenced MI oocytes exhibited notable DNA damage. (**B**) Fold changes in tail moment following *Rad51* RNAi are presented in a bar graph compared to that of the control group. The data are presented as the mean ± SEM. The asterisk represents statistical significance at *p* < 0.05. (**C**) Histogram of oocyte number (frequency) versus tail moment.

*Rad51* plays an essential role in the homologous DNA strand exchange reaction that occurs during recombination and DNA repair. We examined whether depletion of *Rad51* expression affected DNA integrity during oocyte maturation. The comet assay is a useful tool for the detection of single-strand breaks or DSBs in cells. We analyzed DNA damage in ZP-free *Rad51*-silenced oocytes using this assay. Inhibiting Rad51 expression in oocytes significantly increased DNA damage compared with the control oocytes (Fig. 2A,B). A typical histogram shows a rightward-shift in the tail moment of *Rad51* RNAi (Fig. 2C), indicating that *Rad51*-silenced oocytes had more DNA damage. Thus, we concluded that *Rad51*-silencing damaged oocyte DNA and/or mitochondrial DNA (mtDNA), which may have been the main reason that these oocytes went into MI arrest.

### Rad51 depletion disrupted spindle and chromosomal configuration as well as the microtubule-organizing centers (MTOCs)

**Figure 3:**
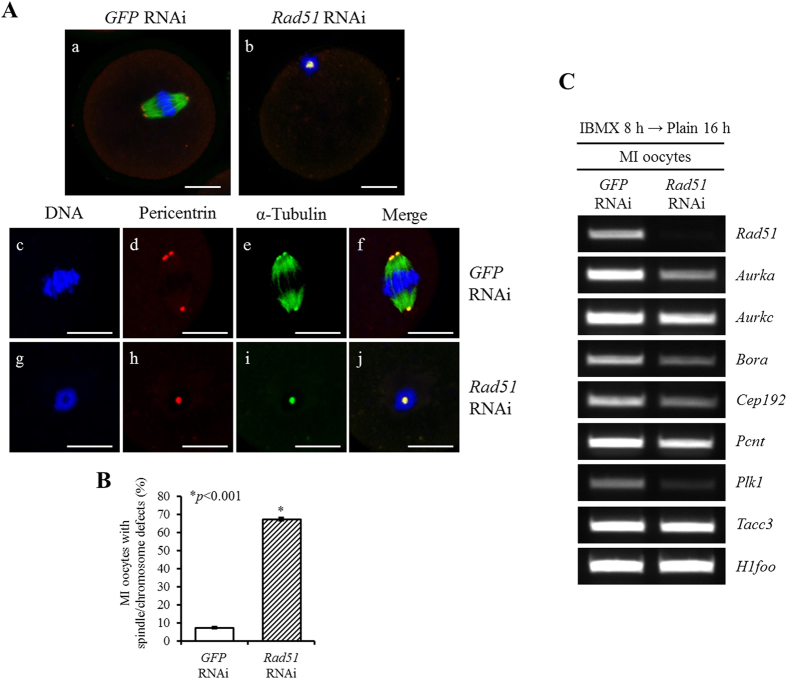
Reduction in *Rad51* expression resulted in defective chromosome segregation and spindle assembly in MI oocytes. (**A**) Immunofluorescence staining for the spindle and chromosomes in MI oocytes after injection with *GFP-* (a,c–f) or *Rad51-* (b,g–j) dsRNA in GV oocytes that were then allowed to mature *in vitro*. The oocytes were fixed in 4% paraformaldehyde and then stained with an antibody against Pericentrin (red) and α-Tubulin (green). DNA was counterstained with DAPI (blue). Lower panel shows the magnified region of the spindle-chromosome complex in another MI oocyte after *GFP* RNAi (c–f) and *Rad51* RNAi (g–j). The scale bars indicate 20 μm. (**B**) Proportion of *GFP* or *Rad51*-dsRNA injected MI oocytes with defects of spindle assembly and chromosome configuration. (**C**) RT-PCR analysis of the expression of MTOC-related genes, including *Aurka*, *Aurkc*, *Bora*, *Cep192*, *Pcnt*, *Plk1* and *Tacc3*, in *Rad51*-silenced MI oocytes. *H1foo* was used as an internal control.

Although GVBD, the resumption of meiotic cell cycle, occurred in the *Rad51*-silenced oocytes, 76.48% of the oocytes could not complete meiosis and arrested at the MI stage. Because the *Rad51*-silenced oocytes failed to complete cytokinesis during oocyte nuclear maturation (Video S2), we evaluated the meiotic spindle structure, the chromosome alignment and the pericentrin distribution in the arrested oocytes using immunofluorescence staining (Fig. 3A). The *GFP* dsRNA-injected control MI oocytes exhibited meiotic spindles with a clear normal barrel shape (Fig. 3Aa,e,f) and well-aligned chromosomes at the metaphase plate (Fig. 3Aa,c,f). Indeed, pericentrin localized to the microtubule-organizing centers (MTOCs) at meiotic spindle poles in *GFP* dsRNA-injected control MI oocytes (Fig. 3Aa,d,f). In contrast, MI spindles were aggregated and intermingled with amassed chromosomes after *Rad51* RNAi treatment (Fig. 3Ab,g–j). In addition, pericentrin localized as only one condensed spot with abnormally aggregated spindles and chromosomes in *Rad51*-depleted MI oocytes (Fig. 3Ab,h,j).

*Rad51* RNAi induced increased proportions of spindle assembly and chromosome configuration defects (Fig. 3B; 7.4% ± 0.5 in *GFP* RNAi vs. 67.5% ± 0.9 in *Rad51* RNAi, *p *< 0.001). Mouse oocytes lack centrioles and possess MTOCs that are composed of pericentriolar material (PCM) proteins. These MTOCs are localized at the poles of the spindles and function as microtubule nucleating centers^20^,^21^,^22^. Because reduced *Rad51* expression led to impairments in spindle assembly and pericentrin, we measured the expression levels of several essential PCM genes and found that six PCM genes (*Aurka*, *Aurkc*, *Bora*, *Cep192*, *Pcnt* and *Plk1,* but not *Tacc3*) were markedly reduced in *Rad51*-silenced MI-arrested oocytes (Fig. 3C).

### Reduced Rad51 expression damaged mitochondrial dynamics and decreased ATP production

**Figure 4:**
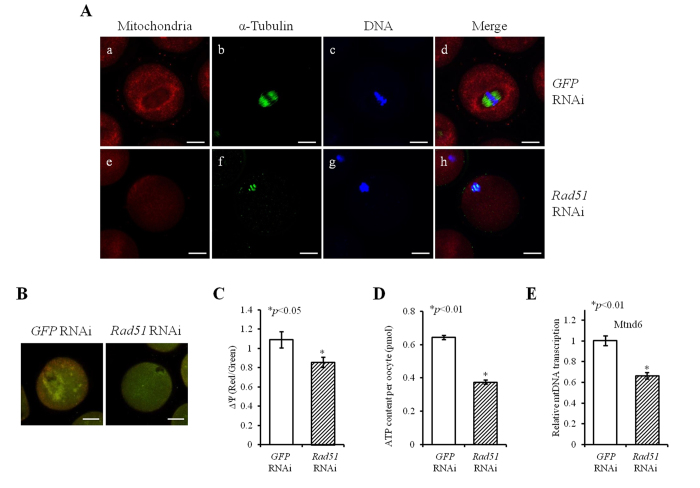
*Rad51* RNAi treatment caused a loss of mitochondrial membrane potential (ΔΨm) and impaired mitochondrial ATP production. (**A**) Abnormal mitochondrial distribution was found in *Rad51*-silenced MI oocytes. *GFP* (a–d) or *Rad51* (e–h) dsRNA was injected into MI oocytes that were precultured in M16 medium containing MitoTracker (mitochondria, red) for 30 minutes and then stained with an antibody against α-Tubulin (green). Cellular DNA was also stained (blue), and the cells were imaged by LSCM. The scale bars indicate 20 μm. (**B**) Effect of *Rad51* on ΔΨm. ΔΨm in *Rad51*-silenced MI oocytes indicated by changes in RITC and FITC intensity, which were measured by LSCM as detailed in the *Materials and Methods* section. The scale bars indicate 20 μm. (**C**) Graphic representation of the results shown in (**B**). The data are presented as the mean ± SEM. The asterisk represents statistical significance at *p* < 0.05 as determined by a paired Student’s *t*-test. (**D**) Effects of *Rad51* down-regulation on oocyte ATP levels. Reduction in *Rad51* expression impaired mitochondrial ATP production. The data are presented as the mean ± SEM. The asterisk represents statistical significance at *p* < 0.01. (**E**) *Rad51* RNAi treatment suppressed mitochondrial *Mtnd6* mRNA expression. The data are presented as the mean ± SEM. The asterisk represents statistical significance at *p* < 0.01.

Because we observed DNA damage and spindle abnormalities, we next quantified the number and distribution of mitochondria in the ooplasm. Mitochondrial quantity was reduced in the ooplasm of *Rad51*-silenced MI oocytes compared to *GFP*-silenced MI oocytes, and the mitochondrial distribution pattern was also changed (Fig. 4A). In the control group, after treatment with *GFP* RNAi, mitochondria were mainly concentrated around the developing meiotic spindle and homogeneously distributed throughout the rest of the ooplasm (Fig. 4Aa–d). Conversely, there was no accumulation of mitochondria around the abnormally aggregated spindles in the *Rad51*-silenced MI oocytes (Fig. 4e–h).

Next, the influence of *Rad51* down-regulation in oocytes on mitochondrial membrane potential (ΔΨm) was measured to validate the abnormalities observed in the mitochondrial number and distribution pattern (Fig. 4B). The ratio between red and green fluorescence was significantly reduced in *Rad51*-silenced oocytes, indicating decreased mitochondrial membrane potential; the difference was statistically significant (Fig. 4C; 1.09 vs. 0.86, *p* < 0.05). We also evaluated the production of mitochondrial ATP in *Rad51*-silenced oocytes. The mean ATP concentrations in *GFP* RNAi oocytes and *Rad51* RNAi oocytes were 0.64 ± 0.013 pmol and 0.38 ± 0.014, respectively, and this difference was statistically significant (Fig. 4D). In addition, the expression of mitochondria-encoded *Mtnd6* transcripts, which are closely associated with mtDNA levels, was significantly decreased in the *Rad51*-silenced oocytes (Fig. 4E). Taken together, *Rad51*-silenced oocytes contain decreased levels of mtDNA, reduced mitochondrial membrane potential and reduced levels of mitochondrial ATP production, which collectively indicate that mitochondria in *Rad51* RNAi oocytes are defective.

### Rad51 depletion induced autophagy-mediated mitophagy of oocytes

**Figure 5:**
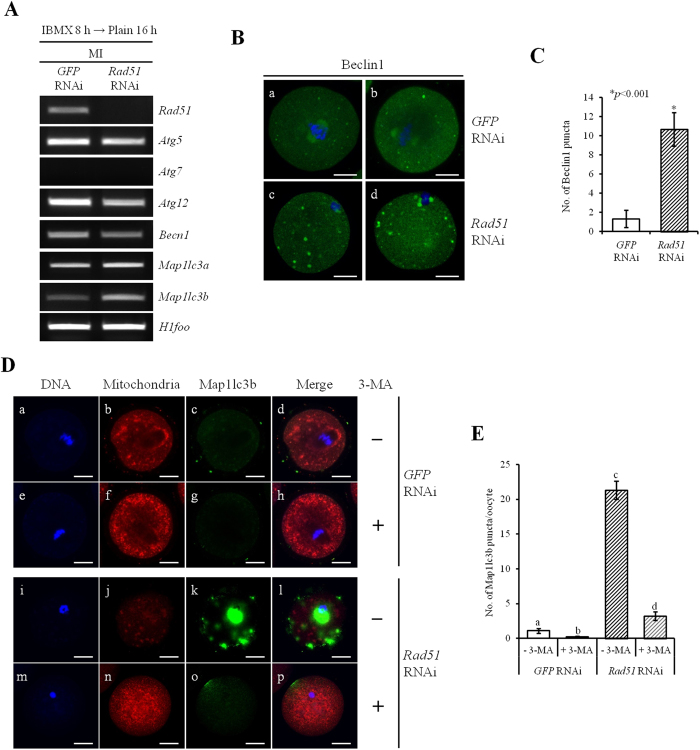
*Rad51* RNAi treatment resulted in the removal of mitochondria by mitophagy. (**A**) The mRNA expression profiles of autophagy-related genes were examined by RT-PCR. The expression levels of *Atg5*, *Atg12* and *Becn1* were slightly reduced, while the expression levels of *Map1lc3a* and *Map1lc3b* were increased in *Rad51*-silenced MI oocytes. *H1foo* was used as an internal control. IBMX 8 h → Plain 16 h, *Rad51*-silenced oocytes cultured in IBMX supplement for 8 hours followed by 16 hours of culture in plain M16 medium. (**B**) Immunofluorescence staining of Beclin1 in MI oocytes. Beclin1 is a marker protein for autophagy initiation. The oocytes were fixed in 4% paraformaldehyde and then stained with an antibody against Beclin1 (green). DNA was counterstained with DAPI (blue). Beclin1 expression is up-regulated in *Rad51*-silenced oocytes. The scale bars indicate 20 μm. (**C**) *Rad51*-silenced MI oocytes displayed increases in the size and number of Beclin1 puncta. The data are the numbers of Beclin1 puncta and are presented as the mean ± SEM. The asterisk represents statistical significance at *p* < 0.001 determined by a paired Student’s *t*-test. (**D**) Representative images showing the formation of Map1lc3b puncta. Map11c3b is a marker protein for mitophagy. Oocytes were fixed in 4% paraformaldehyde and then stained with DAPI (DNA, blue), MitoTracker (mitochondria, red) and anti-Map1lc3b antibody (green) and imaged using the LSCM. The 10 mM 3-MA was used to block autophagy. 3-MA-, oocytes were cultured without 3-MA addition; 3-MA+, oocytes were cultured with 3-MA. The scale bars indicate 20 μm. (**E**) Silencing of *Rad51* expression resulted in the increased formation of Map1lc3b puncta. The average number of Map1lc3b puncta per oocyte was measured in MI oocytes after treatment of *GFP* (a–h) or *Rad51* (i–p) RNAi and cultured in medium supplemented with (e–h, m–p) or without (a–d, i–l) 10 mM 3-MA. The data are presented as the mean ± SEM. Different letters (a~d) indicate significant differences at *p* < 0.001.

Mitophagy is the selective segregation of damaged mitochondria and their subsequent degradation through autophagy^23^. Therefore, we assessed whether the mitochondrial reduction observed in *Rad51*-silenced oocytes was mitophagy-dependent. First, we observed the changes in six autophagy-related genes (*Atg5*, *Atg7*, *Atg12*, *Becn1*, *Map1lc3a* and *Map1lc3b*) via RT-PCR analyses. Among these genes, *Atg7* was not detected in oocytes (Fig. 5A). In *Rad51*-silenced oocytes, the expression levels of three autophagy-related genes, *Atg5*, *Atg12* and *Becn1*, were slightly decreased, while the expression levels of two autophagy-related genes, *Map1lc3a* and *Map1lc3b*, were markedly increased (Fig. 5A).

Beclin1 is a mammalian homolog of yeast autophagy-related gene 6 (*Atg6*) and is essential for autophagy. Induction or phosphorylation of Beclin1 is required to initiate autophagosome formation through the activation of class III phosphoinositide 3-kinase (PI3K)^24^. In *GFP* dsRNA-injected control oocyte, Beclin1 proteins were observed as small spots and were distributed evenly in the ooplasm (Fig. 5Ba,b). However, in *Rad51*-silenced MI oocytes, more and larger Beclin1 protein spots were detected in the ooplasm (Fig. 5Bc–d,C).

Another protein, Map1lc3b, a structural protein in the mitophagosomal membrane, is widely used as a marker of mitophagy^25^. After *Rad51* RNAi treatment, MI-arrested oocytes were analyzed for Map1lc3 localization by counting the number of Map1lc3b puncta throughout the ooplasm. As expected, we found that *Rad51* depletion increased the formation of Map1lc3b puncta compared to the control (Fig. 5Dc–d,k,l,E). When we tested the effects of the autophagy inhibitor 3-methyladenine (3-MA), an inhibitor of autophagy via the inhibition of PI3K activity, on *Rad51* silencing-induced mitophagy, 3-MA selectively blocked autophagosome formation (Fig. 5Dk,l,o,p,E). Consistently, the amount of mitochondria, which was depleted in *Rad51*-silenced oocytes, was increased to levels comparable to those in the control by treatment with 3-MA (Fig. 5Db,j,f,n). These results suggest that Rad51 depletion induces autophagy-mediated mitophagy of oocytes.

### Rad51 safeguards mitochondrial activity in oocytes

**Figure 6:**
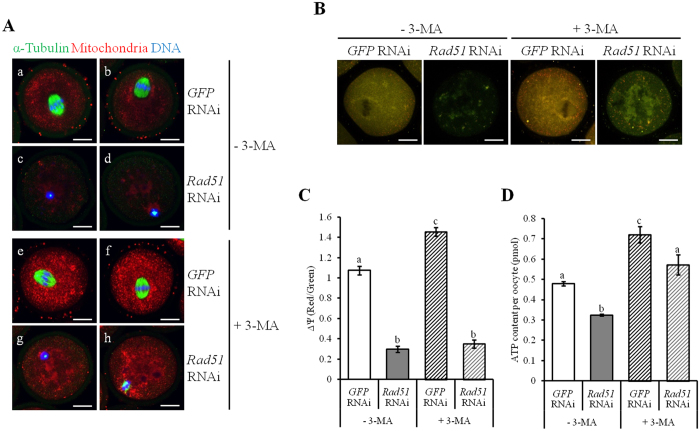
Despite the accumulation of mitochondria by 3-MA, these mitochondria were malfunctional. (**A**) Immunofluorescence staining for spindles and chromosomes after *GFP* (a,b,e,f) or *Rad51* RNAi (c,d,g,h) and cultured without (a–d) or with 10 mM 3-MA (e–h). Oocytes were immunostained for spindles (α-Tubulin; green), mitochondria (MitoTracker; red) and chromosomes (DAPI; blue). The scale bars indicate 20 μm. (**B**) Effect of mitophagy inhibition on ΔΨm after *Rad51* silencing. ΔΨm was measured using JC-1 staining. The ratio of RITC to FITC intensity was calculated. The scale bars indicate 20 μm. (**C**) Quantitative analysis of ΔΨm associated with *Rad51* disruption and mitophagy inhibition is summarized. The data are presented as the mean ± SEM. Different letters (a~c) indicate significant differences at *p *< 0.05. (**D**) Effects of mitophagy inhibition on ATP levels in *Rad51* down-regulated oocytes. ATP levels were measured as described in the Materials and Methods. The data are presented as the mean ± SEM. Different letters (a~c) indicate significant differences at *p* < 0.05.

In Fig. 5, we noted that chromosomal alignment was not rescued by chemical inhibition of autophagy, despite the total number of rescued mitochondria (Fig. 5Da,i,e,m). To further examine whether defective spindle formation could be rescued using a mitophagy inhibitor, *Rad51*-silenced oocytes were treated with 3-MA and then loaded with MitoTracker and immunostained with α-Tubulin. *GFP* dsRNA-injected MI oocytes displayed normal meiotic spindles with aligned chromosomes incubated with (Fig. 6Ae,f) or without 3-MA (Fig. 6Aa,b). Consistent with the chromosome alignment results, disrupted meiotic spindle organization in *Rad51*-depleted oocytes (Fig. 6Ac,d) was not rescued by inhibition of mitophagy (Fig. 6Ag,h). To address whether the mitochondria accumulated in *Rad51*-depleted oocytes upon 3-MA treatment were functional, we examined the ΔΨm and mitochondrial ATP production in *Rad51*-silenced oocytes. We found that mitophagy inhibition did not rescue the decreases in ΔΨm caused by *Rad51* depletion (Fig. 6B,C). Although ATP content was increased by 3-MA both in *GFP* RNAi- and *Rad51* RNAi-treated oocytes, the amount of ATP in *Rad51* RNAi oocytes was statistically lower than that of *GFP* RNAi oocyte (Fig. 6D). These results indicated that the mitochondria accumulated upon 3-MA treatment were not functional; furthermore, treatment also did not rescue the spindles and the chromosomal abnormalities.

**Figure 7:**
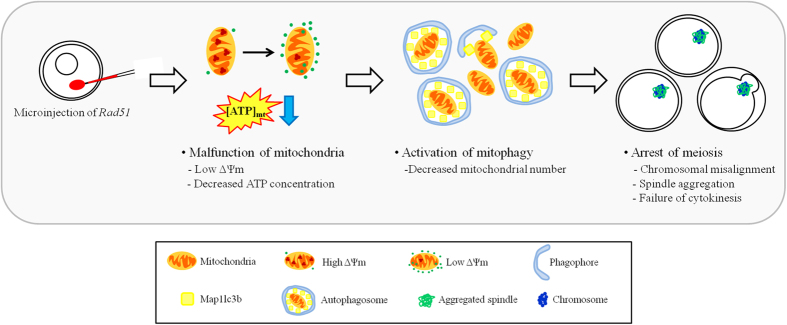
*Rad51* RNAi treatment induced mitochondrial malfunction and mitophagy and subsequently incomplete cytokinesis during oocyte meiosis. *Rad51* silencing-induced mitochondrial malfunction led the decreased ATP production and reduced ΔΨm in oocytes. *Rad51*-silenced oocytes had markedly fewer mitochondria as a result of the activation of mitophagy. In addition, abnormal spindle aggregation and chromosomal misalignment caused by low mitochondrial quality and quantity resulted in cytokinesis failure; as a result, *Rad51*-silenced oocytes were mainly arrested at MI.

Taken together, we concluded that Rad51 is involved in maintaining mitochondrial quantity and quality in mammalian oocytes. *Rad51* depletion induced genome instability in nuclear DNA and/or mtDNA in oocytes, resulting in the dysfunction of mitochondria and the subsequent induction of autophagy-based mitophagy. Mitochondrial depletion caused abnormalities in the MTOCs and spindle formation and defective chromosome organization. These changes resulted in incomplete cytokinesis during meiotic division (Fig. 7).

## Discussion

In the current study, we report a novel role for Rad51 associated with mitochondrial functioning during oocyte maturation *in vitro*. In *Rad51*-silenced MI-arrested oocytes, we observed a decreased quantity of mitochondria and a homogenous distribution of mitochondria throughout the cytoplasm. In contrast, in normal oocytes, mitochondria accumulated around developing meiotic spindles. Consistent with a previous report demonstrating that rearrangement of mitochondria during mouse oocyte maturation is associated with ATP production^26^, the ATP content in the *Rad51*-deficient MI-arrested oocytes was approximately 60% of that in the control oocytes. Furthermore, the mitochondrial membrane potential was also decreased in the *Rad51*-deficient oocytes, and the transcription of the mitochondrial gene *Mtnd6* was approximately 70% of that in the control oocytes. These data suggest that Rad51 functions to maintain both the quantity and the quality of mitochondria in oocytes. It remains to be resolved how *Rad51* depletion causes defects in mitochondrial activity and mitochondrial gene expression during oocyte maturation. Because large amounts of Rad51 are localized in the inner matrixes of mitochondria and because this protein functions to maintain mitochondrial gene copy number^27^,^28^,^29^, one possible mechanism for this process is the direct contribution of Rad51 to sustaining mitochondrial genome stability during oocyte maturation.

In mammalian oocytes, mitochondrial localization is microtubule-dependent^30^,^31^. Thus, the disruption in spindle assembly following *Rad51* depletion, the abnormal distribution of mitochondria, and the reduced number of mitochondria could be all strongly interrelated, in agreement with previous findings that the anatomical association of spindles and mitochondria is linked to meiotic progression in oocytes^30^,^32^.

Recently, global trends have changed as a result of modern lifestyles—for example, women now regularly delay marriage and childbirth until they are in their 30s—which has led to growing problems in maternal reproductive senescence. One hallmark of oocyte aging is an accumulation of genetic mutations in genomes as well as in mtDNA^3^,^12^. Genomic instability and mitochondrial dysfunction in older women are strongly associated with poor reproductive performance and infertility^11^,^31^,^33^,^34^,^35^. Cytoplasmic transfer from young donors into older defective recipient oocytes was developed to treat infertility patients with some success, and the major beneficial cytoplasmic components in such cases are believed to be mitochondria^36^,^37^,^38^,^39^. The heteroplasmy found in children born from this cytoplasmic transfer technique has led to the ethical dilemma of whether such children have three parents and has resulted in moratoriums on ooplasm transfer in some countries^33^. However, a mitochondrial transfer technique developed for couples with mitochondrial diseases could be an exception to this ethical quandary. Nevertheless, we suggest using Rad51 microinjection to rescue reduced oocyte competency related to aging as an alternative to cytoplasmic transfer to avoid heteroplasmy. However, the optimal dose of Rad51 protein to rescue mitochondrial dysfunction in aged oocytes and the safety of this technique must still be resolved before it can be applied in human IVF programs or the somatic cell nuclear transfer technique.

## Methods

### Animals

Imprinting control region (ICR) mice (female and male), exclusively provided by Koatech (Pyeoungtack, Korea), were mated to produce embryos in the breeding facility at the CHA Research Institute of CHA University. All procedures described herein were reviewed and approved by the Institutional Animal Care and Use Committee of CHA University and were performed in accordance with Guiding Principles for the Care and Use of Laboratory Animals.

### Reagents

Chemicals and reagents were obtained from Sigma-Aldrich (St. Louis, MO, USA) unless otherwise noted.

### Oocyte isolation

To isolate GV oocytes, three-week-old female ICR mice were injected with 5 IU pregnant mare’s serum gonadotropin (PMSG) and sacrificed 46 hours later. Isolated ovaries were punctured in M2 medium containing 0.2 mM 3-isobutyl-1-methyl-xanthine (IBMX), and cumulus-enclosed oocyte complexes (COCs) were collected. Cumulus cells were mechanically retrieved from oocytes by repeated extraction through a fine-bore pipette.

To obtain MII oocytes, we injected female mice with 5 IU eCG, followed by 5 IU hCG after 46 hours. MII oocytes were obtained from the oviduct 16 hours after hCG injection. The cumulus cells surrounding the MII oocytes were removed using hyaluronidase (300 U/ml). The isolated murine oocytes were snap frozen and stored at −70 °C prior to RNA isolation.

### Messenger RNA isolation and RT-PCR

Oocyte mRNA was isolated using a Dynabeads mRNA DIRECT kit (Dynal Asa, Oslo, Norway) according to the manufacturer’s instructions. Briefly, oocytes were suspended with lysis/binding buffer and mixed with prewashed Dynabeads oligo dT_25_. After mRNA binding, the beads were washed with buffer A twice, followed by buffer B, and mRNA was eluted with Tris-HCl by incubation at 73 °C. Purified mRNA was used as a template for reverse transcription with an oligo (dT) primer according to the MMLV protocol. RT-PCR was performed with single oocyte-equivalent cDNA and primers (Table 2). Then, the RT-PCR products were separated by electrophoresis on a 1.5% agarose gel and analyzed using a Gel Doc^TM^ EZ Imager (Bio-Rad, Hercules, CA, USA). All experiments were repeated three times.

### Quantitative real-time RT-PCR

Quantitative real-time RT-PCR was performed with single oocyte-equivalent cDNA and *Mtnd6* primers (Table 2), as previously described, using an iCycler iQ™ Detection System (Bio-Rad)^40^. iQ SYBR Green Supermix PCR reagents (Bio-Rad) were used to monitor amplification, and the results were analyzed using iCycler iQ™ proprietary software. The melting curves were used to identify any nonspecific amplification products. The relative expression levels of the target genes were evaluated using the comparative C_T_ method^41^,^42^, and all analytical procedures were repeated at least three times.

### Preparation of Rad51 and GFP dsRNA

For RNAi experiments, we prepared *Rad51* dsRNA. *Rad51*-A (Suppl. Fig. 1A) and *GFP* primers (Table 2) were used to amplify regions of *Rad51* and *GFP* cDNA, respectively, which were then cloned into a pGEM-T Easy (Promega, Madison, WI, USA) vector. After linearization with *Spe*I, each RNA strand was transcribed *in vitro*, annealed at 75 °C and then cooled to room temperature (RT). dsRNA was purified with DNaseI and RNase digestion prior to dilution to 2.9 μg/μl. Purified dsRNA was verified by electrophoresis on a 1% agarose gel, which was used to compare the mobility of dsRNA with that of ssRNA (Suppl. Fig. 1B). *GFP* RNAi was used as an injection control.

### Microinjection and *in vitro* culture

GV oocytes were microinjected with *Rad51* dsRNA in M2 medium containing 0.2 mM IBMX. An injection pipette containing dsRNA solution was inserted into the cytoplasm of an oocyte, and 10 pl of dsRNA was microinjected using a constant flow system (Transjector; Eppendorf, Hamburg, Germany). To determine the rate of *in vitro* maturation, oocytes were cultured in M16 medium containing 0.2 mM IBMX for 8 hours, followed by culture in M16 alone for 16 hours in 5% CO_2_ at 37 °C. After the RNAi experiments were completed, the *in vitro* maturation rates and morphological changes were recorded as previously described^40^. 3-Methyladenine was used to inhibit autophagy. After RNAi, oocytes were cultured in M16 medium containing 10 mM 3-MA.

### Time-lapse video microscopy

Time-lapse video observations were used to track phenotypic changes and the speed of oocyte maturation during *in vitro* culture as previously described^43^. A time-lapse microscope (JuLI^TM^; Digital Bio, Seoul, Korea) was placed in an incubator under 5% CO_2_ at 37 °C, and a culture dish containing oocytes was placed on the microscope stage. Images were automatically captured every 5 minutes for 16 hours for *GFP* RNAi-treated oocytes or 36 hours for *Rad51* RNAi-treated oocytes, and the sequential time-lapse images were converted into movie files using JuLI operation software.

### Western blotting

Protein extracts (150 oocytes per lane) were separated using 12% SDS-PAGE. A PVDF membrane (Amersham Biosciences, Piscataway, NJ, USA) was used to transfer the separated proteins and then blocked for 1 hour in Tris-buffered saline-Tween (TBST) containing 5% non-fat milk. The blocked membranes were then incubated with rabbit anti-Rad51 antibody (1:1000; sc-8349, Santa Cruz Biotechnology, Dallas, TX, USA) or mouse anti-α-Tubulin antibody (1:1000; sc-8035, Santa Cruz Biotechnology) in TBST. After washing in TBST, membranes were incubated with HRP-conjugated anti-rabbit IgG (1:5000; #7074, Cell Signaling Technology, Danvers, MA, USA) or anti-mouse IgG (1:2000; A-2554) in TBST for 1 hour at RT. Bound antibodies were detected using an enhanced chemiluminescence detection system (Amersham Biosciences) according to the manufacturer’s instructions.

### Comet assay

DNA damage was assessed using a neutral version of a comet assay to detect DSBs, as detailed in the manufacturer’s instructions (Trevigen, Gaithersburg, MD, USA). After the zona pellucida (ZP) was removed, oocytes were suspended in 1% low melting point agarose (LMA) at 37 °C, and then the mixture was pipetted onto a pre-coated slide (Trevigen). The slides were incubated at 4 °C in the dark for complete adherence and then immersed in 4 °C lysis solution from Trevigen for 4 hours. The slides were removed from the lysis solution and gently immersed in 4 °C 1X TAE buffer for 30 minutes before being electrophoresed for 40 minutes at 30 V. The slides were immersed in 1 M ammonium acetate at RT for 30 minutes and then immersed in 75% ethanol for fixation at RT for 30 minutes. After the fixation, the slides were incubated at 42 °C for at least 20 minutes until the LMA was fully dried and then stained with 1X SYBR^®^ Green I Staining Solution for 5 minutes. The stained slides were observed under a fluorescence microscope. To quantify individual oocyte comets, analysis was performed using the CASP PC image analysis program according to a previously reported scoring method^44^. We scored each comet according to its tail moment (TM), which is the product of the tail length and the percent tail DNA, TM = TL × (%DNAT).

### Immunofluorescence staining

Denuded oocytes were placed in PBS containing 0.1% polyvinyl alcohol (PBS-PVA), 4% paraformaldehyde, and 0.2% Triton X-100 and then fixed for 40 minutes at RT. After washing in PBS-PVA, fixed oocytes were blocked with 3% BSA-PBS-PVA for 1 hour and then incubated with mouse anti-α-Tubulin antibody (1:100), rabbit anti-Pericentrin antibody (1:100; PRB-432C, BioLegend, San Diego, CA, USA), rabbit anti-Beclin1 antibody (1:100; sc-11427, Santa Cruz Biotechnology), or rabbit anti-Map1lc3b antibody (1:100; ab48394, Abcam, Cambridge, MA, USA) at 4 °C overnight. After washing, the oocytes were incubated with an Alexa Fluor 488- or Alexa Fluor 555-conjugated secondary antibody (Molecular Probes, Eugene, OR, USA) for 1 hour at RT, and DNA was counterstained with DAPI (Promega).

### Staining of mitochondria

To evaluate the distribution of mitochondria, oocytes were stained using MitoTracker Orange CMTMRos (Molecular Probes). MitoTracker, which fluoresces orange, was used at a concentration of 300 nM in M16 supplemented with 0.3% BSA for 30 minutes at 37 °C in the dark. After washing, the oocytes were fixed and immunofluorescently stained with an anti-α-Tubulin antibody and then counterstained with DAPI. The stained oocytes were stored below 4 °C until confocal microscopy was performed. The oocytes were examined using a laser scanning confocal microscope (LSCM; Leica, Wetzlar, Germany). A minimum of 30 oocytes per RNAi group was examined by LSCM.

### Determination of mitochondrial membrane potential (ΔΨm) by JC-1

To measure oocyte mitochondrial activity, ΔΨm assays were performed using the ΔΨm-sensitive fluorescent probe JC-1 (Thermo Fisher Scientific, Waltham, MA, USA). Oocytes were cultured in the dark for 20 minutes in the presence of JC-1 at a concentration recommended by a manufacturer (1 μg/mL) and subsequently imaged on an LSCM using the fluorescein isothiocyanate (FITC, green) and rhodamine isothiocyanate (RITC, red) channels. Two fluorescent images (green and red fluorescence) were acquired in the largest diameter plane of the oocytes. Using confocal software, the signal intensities and the ratio of RITC to FITC for each oocyte were calculated and used as the endpoint for the ΔΨm.

### Measurement of ATP content

The ATP content in 40 oocytes in each group was measured using a Bioluminescent Somatic Cell Assay Kit (FL-ASC) based on the luciferin-luciferase reaction as previously described, with minor changes^45^,^46^. The bioluminescence of each sample was measured using a luminometer (Centro XS3 LB 960; Berthold Technologies, Bad Wildbad, Germany), and an 11-point standard curve (containing 0 to 5.0 pmol ATP) was included in each assay. The ATP content in the oocytes was expressed as pmol/oocyte.

### Statistical analysis

Each experiment was repeated at least three times, and statistical analyses were performed using Student’s *t*-test. Data were derived from at least three separate and independent experiments and expressed as the mean ± SEM. The *p* values were calculated based on a paired *t*-test of the means from the *GFP* RNAi group and the *Rad51* RNAi group, and *p* < 0.05 was considered statistically significant.

## Additional Information

**How to cite this article**: Kim, K.-H. *et al*. The role of Rad51 in safeguarding mitochondrial activity during the meiotic cell cycle in mammalian oocytes. *Sci. Rep.* **6**, 34110; doi: 10.1038/srep34110 (2016).

## Supplementary Material

Supplementary Information

Supplementary Video S1

Supplementary Video S2

## Figures and Tables

**Table 1:** *In vitro* maturation of mouse oocytes after microinjection of *Rad51* dsRNA into GV oocytes.

	Number of oocytes (%)		
	Total	Metaphase I (MI)	Metaphase II (MII)
*GFP* RNAi	158	12 (8.51)	146 (91.49)
*Rad51* RNAi	126	95 (76.48)*	31 (23.52)*

^*^Asterisks indicate significant differences compared to the value of the same stage of the *GFP* RNAi group (paired Student’s *t*-test, *p* < 0.001).

**Table 2:** Primer sequences and RT-PCR conditions.

Gene symbol	Description	Accession numbers	Primer sequences^a^	Annealing temperature	Product size
*Rad51*-A^b^	RAD51 Recombinase	NM_011234.4	For-AAGCTGCTTCAAGGTGGAAT	60 °C	507 bp
			Rev-GAACATGGCTGCTCCATCTA		
*Rad51*-B^c^	RAD51 Recombinase	NM_011234.4	For- AGCTTTCAGCCAGGCAAAT	60 °C	265 bp
			Rev-GCTTCAGCTTCAGGAAGACA		
*Atg5*	Autophagy related 5	NM_053069.5	For- ATGTGCTTCGAGATGTGTGG	60 °C	215 bp
			Rev- CAGGGGTGTGCCTTCATATT		
*Atg7*	Autophagy related 7	NM_001253717.1	For-ATGCCAGGACACCCTGTGAACTTC	60 °C	351 bp
			Rev-ACATCATTGCAGAAGTAGCAGCCA		
*Atg12*	Autophagy related 12	NM_026217.3	For-TTCGGTTGCAGTTTCGCC	63 °C	311 bp
			Rev-CCATGCCTGTGATTTGCAGTA		
*Aurka*	Aurora kinase A	NM_011497.4	For-AGTTGGCAAACGCTCTGTCT	60 °C	160 bp
			Rev-GTGCCACACATTGTGGTTCT		
*Aurkc*	Aurora kinase C	NM_001080965.1	For-ACAACACCGGAACATCCTTC	60 °C	130 bp
			Rev-TGCTGGTCCAACTTCTGATG		
*Becn1*	Beclin1	NM_019584.3	For- TTTGACCATGCAATGGTAGC	60 °C	211 bp
			Rev- TGGTCAGCATGAACTTGAGC		
*Bora*	Bora, aurora kinase A activator	NM_175265.4	For-CCTGGTCCGCATACCTTTTA	60 °C	211 bp
			Rev-CACGGTGTCATTTTCCCTCT		
*Cep192*	Centrosomal protein 192kDa	NM_027556.1	For-AGCTCAACTCGAGCCATCAT	60 °C	142 bp
			Rev-GGGCTCAAAAACTTCAGTGC		
*Map1lc3a*	Microtubule-associated protein 1 light chain 3 alpha	NM_025735.3	For-AGCTTCGCCGACCGCCGTAAG	63 °C	276 bp
			Rev-CTTCTCCTGTTCATAGATGTCAGC		
*Map1lc3b*	Microtubule-associated protein 1 light chain 3 beta	NM_026160.4	For-CGGAGCTTTGAACAAAGAGTG	63 °C	279 bp
			Rev-TCTCTCACTCTCGTACACTTC		
*Mtnd6*	Mitochondrially encoded NADH dehydrogenase 6	NC_005089.1	For-GAGGTTTAGGTTTAATTGTTAGTGGG	60 °C	175 bp
			Rev- CAGTTAGATCCCCAAGTCTCTG		
*Plk1*	Polo-like kinase 1	NM_011121.3	For-TGTAGTTTTGGAGCTCTGTCG	60 °C	148 bp
			Rev-TCCCTGTGAATGACCTGATTG		
*Pcnt*	Pericentrin	NM_008787.3	For- TGGAAAAGCTGTACCTGCAC	60 °C	192 bp
			Rev- TGGTGAAGGGACGTGACATA		
*Tacc3*	Transforming, acidic coiled-coil containing protein 3	NM_001040435.3	For-AAGTACAGCCAGAAAGACCTG	60 °C	299 bp
			Rev-TCCTTCCGCTTCTCAAACC		
*H1foo*	H1 histone family, member O, oocyte-specific	NM_138311	For- GCGAAACCGAAAGAGGTCAGAA	60 °C	378 bp
			Rev- TGGAGGAGGTCTTGGGAAGTAA		
*GFP*^b^	Green fluorescent protein	KF111246.1	For- TGTCCCAATTCTTGTTGAAT	60 °C	561 bp
			Rev- TTGTCTGGTAAAAGGACAGG		

^a^For, Forward; Rev, Reverse.

^b^Primers were used for the preparation of *Rad51* dsRNA and *GFP* dsRNA, respectively.

^c^Different set of primers was used to confirm the knockdown of *Rad51* mRNA after RNAi treatment.
