# Supplementary material for: The role of Rad51 in safeguarding mitochondrial activity during the meiotic cell cycle in mammalian oocytes
Source: Sci Rep. 2016 Sep 28;6:34110. doi: 10.1038/srep34110 (PMC5039699; doi:10.1038/srep34110)

The role of Rad51 in safeguarding mitochondrial activity during the meiotic cell cycle in mammalian oocytes

Kyeoung-Hwa Kim, Ji-Hoon Park, Eun-Young Kim, Jung-Jae Ko, Kyung-Soon Park*, Kyung-Ah Lee*

Institute of Reproductive Medicine, Department of Biomedical Science, College of Life Science, CHA University, Pangyo-Ro 335, Bundang-gu, Seongnam-si, Gyeonggi-do, 463-400, Korea

*Correspondence should be addressed to: Kyung-Ah Lee, leeka@cha.ac.kr or Kyung-Soon Park, kspark@cha.ac.kr

Supplementary Figure 1. Preparation of *Rad51* dsRNA. (**A**) Schematic diagram of the mouse *Rad51* (NM_011234.4) gene showing different locations of PCR-amplified products. *Rad51*_A was used for the preparation of *Rad51* dsRNA, whereas *Rad51*_B was used to confirm the endogenous gene-specific knockdown of *Rad51* after RNAi. (**B**) *In vitro* synthesis of *Rad51* dsRNA. Microphotograph showing sense (S) and antisense (AS) strands after transcription from purified dsRNA, which was used to make *Rad51* dsRNA. MW, molecular weight marker.

Video S1. Time-lapse microscopy of *GFP* dsRNA-injected oocytes. Oocytes showed normal extrusion of the polar bodies during *in vitro* **maturation in the control group.**

Video S2. Time-lapse microscopy of *Rad51* dsRNA-injected oocytes during *in vitro* maturation. The majority of oocytes that could not complete meiosis showed substantial cytoplasmic streaming but were somehow limited to the center area of the oocytes. MI-

arrested oocytes showed increased cytoplasmic fragmentation just prior to the timing of the polar body extrusion but uncompleted the asymmetrical cytokinesis for polar body extrusion even after 36 hours of incubation.

Supplementary Figure 1.

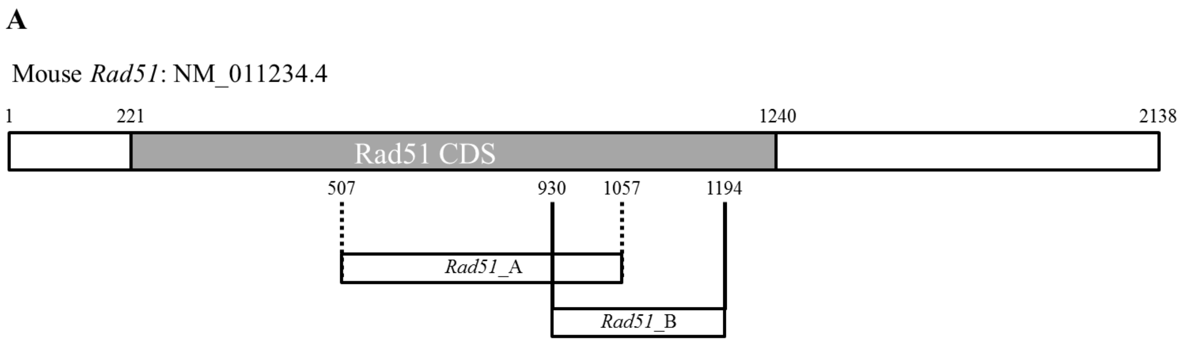

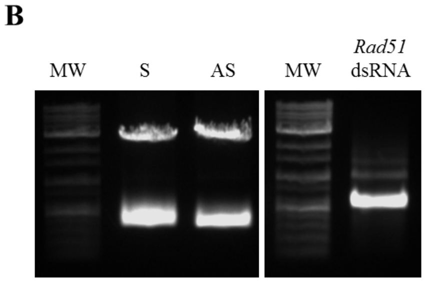

Supplement: Supplementary Information [file srep34110-s1.pdf]
